# Epidemiological Investigation and Analysis of *Orthohantavirus hantanense* and *Orthohantavirus seoulense* From Wild Rodents in Liaoning Province, China

**DOI:** 10.1155/tbed/8897783

**Published:** 2026-05-27

**Authors:** Yijia Wang, Qiqi Liu, Qing Xin, Mingxuan Zhang, Xu Wu, Yongxiang Zhao, Ya Wen, Zimeng Han, Jiaying Li, Jiaxing Wang, Yu Jiang, Xiaohu Han, Feng Jiang, Hongwei Xin

**Affiliations:** ^1^ College of Animal Science and Veterinary Medicine, Shenyang Agricultural University, Shenyang, 110866, Liaoning Province, China, syau.edu.cn; ^2^ Key Laboratory for Infectious Diseases, Ministry of Education, Shenyang Agricultural University, Shenyang, 110866, Liaoning Province, China, syau.edu.cn; ^3^ Manzhouli International Travel Health Care Center, Manzhouli, 021400, Inner Mongolia Autonomous Region, China; ^4^ Academician Workstation for Zoonoses and Biosafety, Shenyang Agricultural University, Shenyang, 110866, Liaoning Province, China, syau.edu.cn; ^5^ Shenyang Center for Disease Control and Prevention, Shenyang, 110623, Liaoning Province, China; ^6^ The Sixth People’s Hospital of Dandong, Dandong, 118002, Liaoning Province, China; ^7^ Qingdao Harwars Biology Group Co., Ltd., No. 23 Wangjiang Road, West Coast New Area, Qingdao, 266555, Shandong Province, China

**Keywords:** hemorrhagic fever with renal syndrome, metatranscriptomics, *Orthohantavirus*, *Orthohantavirus hantanense*, *Orthohantavirus seoulense*

## Abstract

**Background:**

Liaoning Province possesses distinct ecological characteristics, including high forest cover and developed agriculture, which provide suitable habitats for various rodent species. Over the years, hemorrhagic fever with renal syndrome (HFRS), caused by members of the genus *Orthohantavirus*, such as *Orthohantavirus hantanense* (HTNV) and *Orthohantavirus seoulense*, remains highly prevalent in Liaoning Province, representing a significant public health concern. The transmission of these viruses is sustained by wild rodent reservoirs, necessitating continuous surveillance of infection in these host populations. This study is designed to investigate the distribution and genetic evolution of *Orthohantavirus* infections within wild rodent reservoir populations in Liaoning Province from 2022 to 2023 and to provide a scientific basis for effective prevention and control strategies.

**Methods:**

We conducted an epidemiological investigation of 403 wild rodents collected across Liaoning Province. Samples were screened using a combination of metatranscriptomic next‐generation sequencing (mNGS) and specific two‐step RT‐PCR protocols.

**Results:**

We identified a total of 19 *Orthohantavirus*‐positive rodent samples (4.71% prevalence). Initial screening based on national standards (SN/T 2777‐2011) identified 15 positive samples, primarily from the Fushun, Shenyang, and Chaoyang areas. Notably, while standard RT‐PCR failed to detect viral RNA in certain samples from the Dandong area, mNGS revealed HTNV nucleic acid sequences, indicating significant genomic divergence in the primer‐binding regions of the M segment. Consequently, based on the obtained M‐segment sequences, we designed novel, strain‐specific primers (HTN‐DD), which successfully identified an additional four HTNV‐positive samples in the Dandong area. Sequence alignment demonstrated high sequence identity with major strains found in local human infections among these rodent‐derived sequences.

**Conclusions:**

HTNV and SEOV exhibit a significant prevalence in wild rodents across Liaoning, which confirms the region remains a high‐risk area for HFRS. Our findings suggest that viral genomic divergence can compromise standard molecular detection. Therefore, surveillance protocols must be updated to avoid false negatives in clinical diagnosis and epidemiological investigations.

## 1. Introduction

Numerous members of the genus *Orthohantavirus* (family Hantaviridae, order Bunyavirales) are significant zoonotic pathogens causing hantavirus pulmonary syndrome (HPS) and hemorrhagic fever with renal syndrome (HFRS) [1]. The former syndrome is primarily caused by New World *Orthohantaviruses* such as Sin Nombre virus (SNV), whereas the latter is predominantly induced by Old World *Orthohantaviruses*, including *Orthohantavirus hantanense* (HTNV) and *Orthohantavirus seoulense* [2]. The clinical progression of HFRS is classically characterized by five distinct phases: febrile, hypotensive shock, oliguric, polyuric, and convalescent [3]. However, clinical manifestations in patients may deviate from this classical pattern. In severe cases, overlapping phases may occur, whereas milder presentations often exhibit indistinct stage demarcation or omission of specific phases [4]. The severity of the HFRS is largely determined by the viral species involved in the infection, with HTNV inducing more severe clinical damage than SEOV [5, 6].

Members of the genus *Orthohantavirus* comprise enveloped, segmented, single‐stranded, negative‐sense RNA viruses with a virion diameter of ~120–160 nm [7]. Their genomic RNA is organized into three distinct segments designated as S (small), M (medium), and L (large), measuring ~1.8, 3.7, and 6.5 kb in length, respectively. The S segment primarily encodes the nucleoprotein (NP), which complexes with viral nucleic acids to form a nucleocapsid structure essential for viral replication. The M segment encodes the precursor glycoprotein (GPC), which is processed into the envelope GPCs Gn and Gc. These GPCs mediate receptor binding and host cell entry and are the major targets for host neutralizing antibodies. The L segment encodes RNA‐dependent RNA polymerase (RdRp), a crucial enzyme required for both genomic RNA replication and transcriptional synthesis of viral mRNAs [8–10]. Due to its essential biological functions, the M segment contains highly conserved regions flanking the variable domains, making it an ideal target for molecular detection and genotyping. However, notable genetic variability within specific loci can alter primer‐binding sites, potentially compromising the sensitivity of established RT‐PCR assays.

Diseases caused by *Orthohantavirus* members exhibit global endemicity. HPS has been predominantly reported in the Americas since 1993, with thousands of cases documented across Canada, the United States, Brazil, and Argentina, exhibiting a case fatality rate as high as 40% [11, 12]. In China, HFRS poses a serious challenge to public health security, with a widespread impact, as cases have been reported in all provinces. The country accounts for over half of global HFRS cases annually, maintaining the highest reported incidence worldwide [13, 14]. The disease demonstrates distinct seasonal bimodal peaks: the winter peak (November to January) exhibits higher incidence and is primarily caused by HTNV, whereas the spring peak (March–May) is predominantly associated with SEOV [15]. These epidemiological variations are generally attributed to both ecological and anthropogenic factors. Ecological drivers of *Orthohantavirus* transmission include reservoir host dynamics (e.g., population density), landscape features (e.g., forest fragmentation), and climatic conditions (e.g., precipitation seasonality). Anthropogenic influences encompass economic/agricultural activities, land‐use patterns, urbanization processes, and policy implementations, all of which collectively modulate disease emergence in human populations [14, 16–18].

The transmission and maintenance of members of the genus *Orthohantavirus* are characterized by a highly specific coevolutionary relationship with their natural reservoirs, where distinct viral genotypes are typically hosted by specific small mammal species [19]. In China, *Apodemus agrarius* (the striped field mouse) serves as the primary reservoir for HTNV, predominantly inhabiting agricultural landscapes and rural fields [20]. Conversely, *Rattus norvegicus* (the brown rat) is the principal carrier of SEOV [21], exhibiting synanthropic behavior and residing primarily in urban residential areas alongside human habitations. Liaoning Province represents a critical HFRS endemic focus in Northeast China, where these primary reservoir species occupy distinct ecological niches. The viral ecology is further complicated by the identification of variants in secondary hosts, such as *Microtus fortis* (reed vole). To elucidate these complex evolutionary dynamics, we conducted a comprehensive survey of 403 wild rodents across the province to characterize the genetic diversity of circulating HTNV and SEOV strains.

Northeastern China, particularly the eastern Changbai Mountain region, has emerged as a high‐risk area for HFRS, with rising incidence and mortality rates in recent years. To address this public health concern, we collected 403 wild rodent specimens from Liaoning Province for viral screening. Initial epidemiological investigations for HTNV and SEOV were conducted strictly following the SN/T 2777‐2011 industry standard [22]—a protocol published by the former General Administration of Quality Supervision, Inspection and Quarantine of the People’s Republic of China (with entry–exit quarantine responsibilities now under the General Administration of Customs) and specifically designed for disease screening and health quarantine at customs entry–exit ports. Metatranscriptomic sequencing revealed significant nucleotide variations at critical primer‐binding loci in local viral strains, suggesting reduced sensitivity of the standard assay for current variants. To address this limitation, novel primers targeting the HTNV M segment (GenBank: PP438579/PX753259), selected for its genetic diversity and role in encoding GPCs essential for viral infectivity and pathogenicity, were designed based on metatranscriptomic data and applied to retest all specimens. These findings underscore the necessity of updating molecular surveillance tools in light of viral evolution and provide actionable insights for optimizing animal disease surveillance and strengthening public health preparedness against HTNV and SEOV.

## 2. Materials and Methods

### 2.1. Sample Collection and Processing

Wild rodent samples were collected from across Liaoning Province from 2022 to 2023, with a focus on key HFRS‐endemic regions, including Dandong, Fushun, and Chaoyang, as well as the provincial capital, Shenyang. A total of 403 rodents were captured using cage traps and morphologically identified. Subsequently, the rodents were euthanized via CO_2_ asphyxiation (99.5% purity at a flow rate of 200 mL/min) until no vital signs were detected for at least 5 min. Specifically, lung and kidney tissues were collected and divided into two portions. One portion was subjected to initial antigen and nucleic acid screening (RT‐PCR), while the other portion was pooled for metatranscriptomic sequencing analysis. All samples were aliquoted and cryopreserved at −80°C. This study was approved by the Institutional Animal Care and Use Committee (IACUC Protocol Number 2021040701).

### 2.2. Viral RNA Extraction

Total RNA was extracted from ~30 mg of tissue using the FastPure Viral DNA/RNA Mini Kit (Vazyme Biotech Co., Ltd., Nanjing, China) strictly following the manufacturer’s instructions. RNA concentration and purity were assessed using a NanoDrop 2000 spectrophotometer (Thermo Fisher Scientific, USA), ensuring an A260/A280 ratio between 1.8 and 2.1.

### 2.3. Molecular Detection

HTNV and SEOV were detected using a standard two‐step nested RT‐PCR protocol (Table 1) based on SN/T 2777‐2011. First, cDNA was synthesized using the HiScript III 1st Strand cDNA Synthesis Kit (+gDNA wiper) (Vazyme). Subsequently, PCR amplification was performed in a 20 μL reaction mixture containing 10 μL of 2 × Taq Plus Master Mix II (Dye Plus), 6.4 μL of ddH_2_O, 0.8 μL of each primer (10 μM), and 2 μL of template (cDNA for the first round; first‐round product for the second). The thermal cycling conditions were as follows: initial denaturation at 94°C for 5 min; 35 cycles of 94°C for 30 s, 54°C for 20 s, and 72°C for 30 s, followed by a final extension at 72°C for 10 min. Amplified PCR products were visualized using agarose gel electrophoresis. Positive amplicons were sequenced by Sangon Biotech (Shanghai) Co., Ltd.

**Table 1:** Primer sequences for the detection of HTNV and SEOV.

Target	Primer name	Sequence (5′–3′)	Length (bp)
Genus‐universal	OUTF	AAAGTAGGTGITAYATCYTIACAATGTGG	—
	OUTR	GTACAICCTGTRCCIACCCC	
HTNV	HTNV‐F	GAATCGATACTGTGGGCTGCAAGTGC	383
	HTNV‐R	GGATTAGAACCCCAGCTCGTCTC	
SEOV	SEOV‐F	GTGGACTCTTCTTCTCATTATT	287
	SEOV‐R	TGGGCAATCTGGGGGGTTGCATG	

*Note:* OUTF, outer forward primer; OUTR, outer reverse primer; F, forward primer; R, reverse primer. The symbol “I” denotes inosine, and “Y” (C/T)/“R” (A/G) represent mixed bases. The genus‐universal primers (OUTF/OUTR) target the M segment (GPC gene) and were synthesized strictly according to the SN/T 2777‐2011 industry standard.

### 2.4. Acquisition of HTNV and SEOV Nucleic Acid Sequences

To investigate potential genomic variations in samples that tested negative or weakly positive by standard methods, 100 rodent samples were selected for metatranscriptomic next‐generation sequencing (mNGS) using a strategy that combined random sampling in high‐density areas with comprehensive inclusion in low‐density regions. RNA libraries were constructed and sequenced on the Illumina NovaSeq X Plus platform (HongweiST, Inc., Beijing) to generate 150 bp paired‐end reads. Raw reads were filtered to remove host (rodent) genome sequences and low‐quality data. The remaining nonhost reads were assembled de novo and aligned against reference *Orthohantavirus* genomes to reconstruct full‐length viral sequences.

### 2.5. Reconstruction and Validation of HTNV Detection Method

mNGS analysis of the pooled samples successfully identified HTNV sequences that were previously missed by the SN/T 2777‐2011 protocol. Multiple sequence alignment revealed four nucleotide mismatches between the standard primers and the local HTNV M‐segment variants (GenBank: PX753259), explaining the false‐negative results. To address the detection gap, we designed the HTN‐DD primer set to preferentially target the specific M‐segment signatures of the Dandong variants. BLAST verification confirmed that the primer sequences are highly conserved within the local isolates, providing a tailored detection solution that outperforms the generic standard primers for these specific variants. To validate the efficacy of the newly designed primers, the HTN‐DD set was applied to rescreen the four samples that showed discordant results between the standard SN/T 2777‐2011 protocol and mNGS.

### 2.6. Genetic Evolutionary Analysis of HTNV and SEOV

The HTNV and SEOV gene sequences obtained via metatranscriptomic sequencing were compiled into a FASTA file alongside reference strains downloaded from GenBank and the corresponding sequences from domestic regions using ClustalW in MEGA 11 software. Saturation testing was performed using the DAMBE software to ensure phylogenetic signal stability. All sequences were aligned, trimmed to uniform lengths, and analyzed for genetic distances using the MEGA 11 software. A neighbor‐joining (NJ) phylogenetic tree was constructed with the following parameters: bootstrap method (1000 replicates) for phylogeny testing, pairwise deletion for gaps/missing data treatment, and default settings for other options. The complete coding sequences (CDSs) of the representative strains identified in this study have been deposited in GenBank under Accession Numbers PX753258–PX753260 (HTNV S, M, L segments) and PX753261–PX753263 (SEOV S, M, L segments).

## 3. Results

A total of 403 rodent samples were initially screened using the standard SN/T 2777‐2011 protocol. SEOV‐positive samples were identified, whereas no HTNV‐positive samples were detected. However, metatranscriptomic sequencing revealed the presence of HTNV nucleic acid sequences. Based on these findings, primers were redesigned for targeted detection, and phylogenetic analysis was performed using the CDS regions of the nucleic acids.

### 3.1. Detection Results by Standardized Protocol

**Figure 1:**
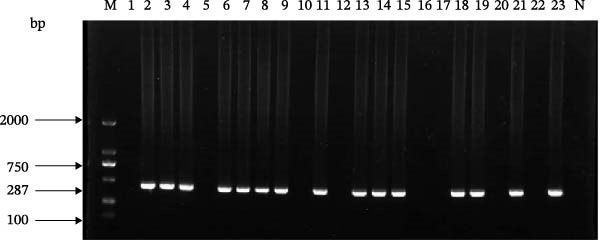
Results of SEOV RT‐PCR detection in field rodent samples from Liaoning Province. *Note:* M: DNA marker DL2000; N: negative control; Samples 2, 3, 4, 6, 7, 8, 9, 11, 13, 14, 15, 18, 19, 21, and 23 were positive for SEOV.

Fifteen samples (3.72%) tested positive for SEOV (Figure 1), predominantly distributed in Fushun, Shenyang, and Chaoyang regions (Table S1). Notably, no HTNV‐positive samples were detected using the standardized protocols, even in the Dandong border area where *A. agrarius* is abundant.

### 3.2. Identification of False Negatives and Primer Optimization

**Figure 2:**
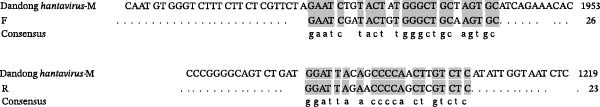
Alignment of primer sequences from the industry‐standard detection methods with corresponding HTNV sequences identified in this study.

To investigate the absence of HTNV detection, 100 representative samples were pooled and subjected to mNGS. Analysis revealed the presence of HTNV nucleic acid sequences within the pooled samples, confirming the actual presence of HTNV in these samples and indicating that the virus was indeed present. To understand why the initial screening failed to detect the virus, we extracted these mNGS reads for further analysis. Sequence alignment identified significant nucleotide mismatches at the binding sites of the standard primers (Figure 2). As illustrated in the alignment, multiple base mutations were observed in the viral sequences compared to the standard primer sequences, explaining the detection failure. The primer sequences from the industry‐standard detection method were aligned with the corresponding fragments obtained via metatranscriptomic sequencing using the DNAMAN software. Comparative analysis revealed the alignment of standard SN/T 2777‐2011 primers with the local HTNV M segment sequences, highlighting four nucleotide mismatches (indicated by red arrows) that likely caused the false‐negative results (Figure 2). Here, “F” and “R” denote the primer sequences specified in the industry‐standard protocol, while “Dandong *hantavirus*‐M” refers to the corresponding sequence identified through metatranscriptomic sequencing.

Based on the HTNV M segment sequences derived from the metatranscriptomic data, a new primer pair (HTNV‐DD‐F: 5′‐GGAATTGCAGAGGGAAATCAAT‐3′; HTNV‐DD‐R: 5′‐ATCCAGGTGGTTTAAGTCAAGA‐3′) was designed to amplify a 310 bp fragment.

**Figure 3:**
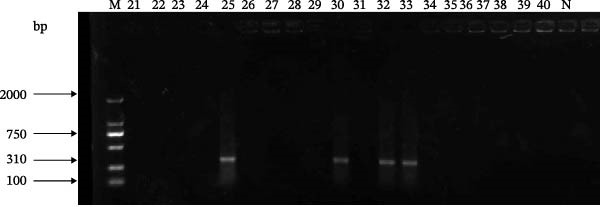
RT‐PCR results of HTNV in wild rodent samples from Liaoning Province. *Note:* M: DNA marker DL2000; N: negative control; Samples 25, 30, 32, and 33 were positive for HTNV.

Using these newly designed primers, we retrospectively screened the entire cohort of all 403 samples, which successfully identified a total of four HTNV‐positive samples. The electrophoresis results confirmed specific amplification with clear bands at the expected 310 bp size (Figure 3). These four positive samples all originated from Dandong (Tongyuanpu Town and Jingu Village). Combining the results, the total *Orthohantavirus* prevalence was 4.71% (19/403).

### 3.3. Epidemiology and Host Distribution of HTNV and SEOV

Wild rodent samples in this study were predominantly collected from Fengcheng (Dandong) and Fushun in Liaoning Province, with fewer samples from other regions. Among the tested samples, 15 SEOV‐positive rodents were identified, which were primarily distributed in Fushun (six samples, 5.88%), Shenyang (eight samples, 8.08%), and Chaoyang (one sample, 25%). Within Shenyang, positive detections were concentrated in the Heping District (two samples, 18.18%), Xinmin City (four samples, 9.30%), and Yuhong District (two samples, 10%). Additionally, four HTNV‐positive samples were detected, located in Jingu Village, Tangshi Town, Zhenxing District, Dandong (one sample, 3.70%) and Tongyuanbao, Fengcheng City (three samples, 2.46%) (Table S1).

The wild rodent species collected in Liaoning Province included *A. agrarius* (striped field mouse), *Mus musculus* (house rodents), and *R. norvegicus* (brown rat). Among these, *R. norvegicus* (*n* = 283) constituted the dominant species, accounting for 70.22% of all samples. SEOV‐positive samples were exclusively identified in *R. norvegicus* (*n* = 15, 5.30%). In contrast, HTNV positivity was detected in three *A. agrarius* (4.17%) specimens, consistent with its typical host. Notably, a single *M. musculus* (2.08%, 1/48) also tested positive for HTNV. Complete data regarding sample distribution and positivity rates are provided in Table S1.

### 3.4. Genetic Evolutionary Analysis

#### 3.4.1. Genetic Evolutionary Analysis of HTNV

Near‐full‐length sequences of the HTNV S, M, and L segments were obtained (GenBank Accession Numbers PX753258–PX753260). A total of 75 members of the Hantaviridae family were retrieved from the latest virus taxonomy list in the official ICTV database. Valid S (*n* = 51), M (*n* = 66), and L (*n* = 21) nucleic acid sequences were downloaded, along with HTNV sequences from domestic regions in China. NJ trees were constructed using MEGA 11 software (Figure 4A–C). In the S‐segment phylogenetic tree, the Dandong strain clustered closely with the HTNV strain DandongHu‐31 isolated from Dandong, forming a distinct branch. For the M segment, the Dandong strain grouped with isolates from Jilin Province in a minor clade. In the L‐segment tree, sequences closely related to Dandong primarily originated from Fuyuan City (Heilongjiang Province) and the adjacent Khabarovsk region of Russia. Comparative analysis revealed that the Dandong strains belonged to the classical HTNV lineage.

Figure 4Genetic evolutionary analysis. (A) HTNV S segment, (B) HTNV M segment, (C) HTNV L segment, (D) SEOV S segment, (E) SEOV M segment, and (F) SEOV L segment.

**Figure:**
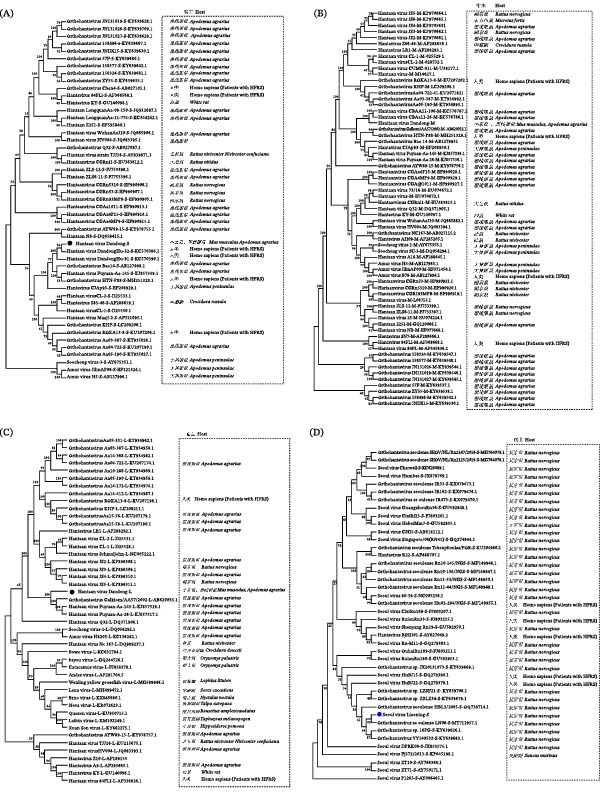

**Figure:**
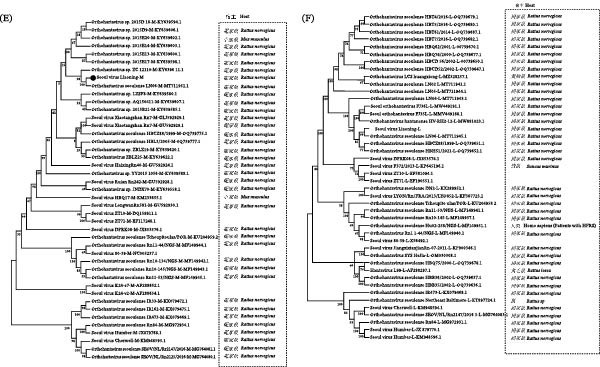

#### 3.4.2. Genetic Evolutionary Analysis of SEOV

Near‐full‐length sequences of the SEOV S (1661 bp), M (3599 bp), and L (6220 bp) segments were obtained (GenBank Accession Numbers PX753261–PX753263). For phylogenetic analysis, this study was guided by national standard literature, and we downloaded the sequences of S, M, and L fragments of Seoul virus (SEOV) for the construction of a phylogenetic tree. The downloaded sequences were intended to provide a broad comparative background covering representative strains from several major outbreak origins in China, including Shandong, Yunnan, Jilin, Jiangxi, and South China, which were selected with reference to several published regional and national studies [21–27], and NJ trees were constructed using the aforementioned method (Figure 4D–F). The topological structures of the phylogenetic trees for all the three segments (S, M, and L) were largely consistent. Phylogenetic analysis revealed that the SEOV sequences clustered most closely with Liaoning isolate LN06 across all segments, forming a unified branch. Host infection rate analysis indicated that *R. norvegicus* (brown rat) had a markedly higher infection rate than the other hosts. All sequences within the same clade as that of Liaoning were exclusively derived from *R. norvegicus*.

## 4. Discussion

HFRS caused by HTNV and SEOV remains a major public health concern in China because of its high morbidity and mortality rates [23]. Although cases have been reported globally, the vast majority (~90%) occur in China. Since HFRS is a prioritized notifiable infectious disease in China, comprehensive prevention and control measures—including disease surveillance, rodent control, environmental remediation, and vaccination programs—have significantly reduced HFRS incidence rates. Nevertheless, over the past decade, ~10,000 HFRS cases have been reported annually, posing a persistent threat to public health [24]. Liaoning Province, an HFRS‐endemic region, experiences recurrent outbreaks and sporadic cases annually [25]. Notably, a marked seasonal surge in human infections occurs between October and December, coinciding with agricultural activities such as crop harvesting, drying, and storage. During this period, heightened rodent activity and crop contamination by rodent excreta contribute to the transmission of HFRS, reflecting its distinct epidemiological seasonality. Continuous surveillance is therefore imperative in these high‐risk municipalities across Liaoning Province [26].

Liaoning Province exhibits high summer precipitation and substantial forest coverage, providing favorable natural environments for the survival and reproduction of rodents. These areas harbor a wide distribution of vector organisms, including rodents and ticks, historically maintaining high prevalence of multiple vector‐borne infectious diseases. In contrast, western Liaoning features lower forest coverage with reduced population density and smaller wildlife density, resulting in relatively low specimen collection numbers. Consistent with historical data, dominant rodent species in Liaoning include *R. norvegicus*, *Mus musculus*, and *A. agrarius*. In this study, these three species were sampled in eastern Liaoning, with *R. norvegicus being* numerically predominant, followed by *A. agrarius and M. musculus*. Despite the presence of these reservoir hosts, HTNV was not detected using the SN/T‐2777‐2011 industry standard protocol. Comparative analysis of primer sequences from the SN/T‐2777‐2011 protocol and corresponding genomic segments obtained via metatranscriptomic sequencing revealed multiple nucleotide variations, predominantly mismatches at the primer binding sites. These genetic alterations have driven the emergence of novel HTNV genotypes, serving as a primary mechanism for viral evolution, which may correlate with shifts in virulence and infectivity.

Our epidemiological data indicate that the detection rate of SEOV (3.72%) in wild rodent samples from Liaoning Province was relatively higher than that of HTNV (0.99%), suggesting a slightly elevated risk of SEOV‐associated infection compared with HTNV. However, HTNV typically induces more severe clinical manifestations of HFRS than SEOV. Notably, since 2021, the incidence of severe and critical HFRS cases in northeastern Liaoning has increased significantly, often exhibiting temporal clustering. For instance, between late November and early December 2021, over 10 patients with severe or critical HFRS were admitted to a regional infectious disease hospital in Liaoning Province. Serological testing was used only as a preliminary screening tool for anti‐*Orthohantavirus* antibodies, and cross‐reactivity among *Orthohantaviruses* (including HTNV and SEOV) may occur. Therefore, all suspected positives were further confirmed and typed by molecular methods (RT‐PCR and sequencing) to ensure species‐level identification [27]. Beyond individual host susceptibility, genetic evolution of the local virus strains may partially explain this surge in severity. Specifically, the nucleotide mutations identified at the primer‐binding sites (as detailed in our results) corresponded to two specific amino acid substitutions (IYLVGCKC → IDTVGCKC) in the viral GPC. Whether these substitutions alter the viral pathogenicity or transmissibility requires further investigation. Furthermore, our laboratory has confirmed the prevalence of SFTSV genotypes in this region [28]. Given the overlapping endemicity with Hantavirus, potential coinfection may contribute to some severe or critical HFRS cases, warranting further investigation. Thus, precise diagnostic and therapeutic strategies should be implemented in clinical settings to address potential coinfections.

Rodents infected with HTNV typically exhibit no overt clinical symptoms, yet persistently harbor and shed the virus over extended periods. Viral shedding occurs via urine, feces, and saliva, thereby contaminating the environment. Human infections arise through exposure to contaminated food, inhalation of virus‐laden aerosols, or bites from infected rodents. Epidemiologic and etiologic studies have confirmed the circulation of two *hantavirus* genotypes in China: HTNV (carried predominantly by *A. agrarius*) and SEOV (hosted primarily by *R. norvegicus*). Although HTNV is generally considered to exhibit relatively strict host specificity, we detected an HTNV‐positive sample in *Mus musculus*, which is most consistent with a sporadic cross‐species transmission (spillover) event in the Dandong region. The phylogenetic placement of the *M. musculus*‐derived sequence within the local geographic HTNV lineage is consistent with geographically structured circulation with occasional spillover, rather than evidence of broad loss of host specificity or host adaptation. Such host diversification amplifies the complexity and unpredictability of viral transmission dynamics. Notably, the habitats of *A. agrarius* (predominantly wild/agricultural) and *M. musculus* (commensal/transitional) frequently overlap in this region. Close interspecies interactions between these rodents may facilitate cross‐species viral transmission, raising concerns regarding potential genetic recombination or reassortment events.

As an RNA virus, HTNV is prone to genetic mutations, which may enable evasion of existing detection methods. The primers in the SN/T 2777‐2011 industry standard were designed based on the conserved sequences of HTNV. However, the genetic diversity of HTNV may lead to an incomplete matching between specific strains and primer sequences. Such mismatches could reduce the detection sensitivity and specificity, significantly impacting epidemic surveillance and individual diagnosis. Bioinformatics alignment using MEGA 11 between the national standard primers and viral M segment sequences revealed nucleotide variations in HTNV. To address viral sequence diversity, detection methods require continuous optimization to improve coverage. This includes designing multiple primer sets targeting distinct viral variants and developing broad‐spectrum detection methods adaptable to sequence variations. Future research should prioritize the systematic evaluation of HTNV genetic diversity and development of detection technologies tailored to this diversity. Additionally, studies on viral evolution are critical for preventing and controlling HFRS transmission. In conclusion, the observed mismatches between the national standard primers and HTNV sequences underscore the impact of viral genetic diversity on disease diagnosis and control strategies. In‐depth investigation into these mismatches and their underlying mechanisms will provide a scientific foundation for improving disease surveillance, diagnostic technologies, and the development of vaccines and therapeutics. Given declining costs and technical maturation of mNGS, we propose its periodic integration into hantavirus surveillance in Liaoning Province. This strategy mitigates detection gaps in the port‐quarantine standard (SN/T 2777‐2011) caused by viral sequence divergence, enhances identification of established and emerging variants, and provides high‐resolution genomic data to strengthen transboundary disease early warning and precision public health responses.

## Author Contributions

Xiaohu Han, Xu Wu, and Hongwei Xin conceptualized the research hypothesis and designed the overall study protocol. Feng Jiang, Xiaohu Han, Xu Wu, Yongxiang Zhao, Mingxuan Zhang, Ya Wen, Jiaxing Wang, and Yu Jiang performed sample collection and molecular experiments. Qiqi Liu, Xiaohu Han, Qing Xin, Zimeng Han, and Jiaying Li contributed to data curation and organization. Yijia Wang, Qiqi Liu, and Xiaohu Han edited the tables. Literature retrieval was conducted by Xiaohu Han, Yijia Wang, and Qiqi Liu, who also participated in manuscript writing. Xiaohu Han, Qiqi Liu, Feng Jiang, and Yijia Wang performed statistical analysis and drafted the initial manuscript. Xiaohu Han, Feng Jiang, and Hongwei Xin critically reviewed and revised the original manuscript. All authors have contributed significantly to data acquisition, analysis, interpretation, and manuscript revision and editing.

## Acknowledgments

During the preparation of this work the authors used DeepSeek in order to translate the original manuscript.

## Funding

This work was supported by the Research Program of the General Administration of Customs (Grant 2025HK006) and the Science and Technology Partnership Program of the Ministry of Science and Technology of China (Grant KY201901014).

## Disclosure

After using DeepSeek, the author reviewed and edited the content as needed and takes full responsibility for the content of the published article.

## Ethics Statement

This protocol was approved by the Shenyang Agriculture University Animal Ethics Committee (Letter Number 2021040701) after rigorous review of its necessity and welfare safeguards.

## Conflicts of Interest

The authors declare no conflicts of interest.

## Supporting Information

Additional supporting information can be found online in the Supporting Information section.

## Supporting information


**Supporting Information** One supporting information is provided: Table S1: Detailed information on sampling sites, sample size, and positive numbers and positive rates of HTNV and SEOV in wild rodents in Liaoning Province.

## Data Availability

The data that support the findings of this study are available upon request from the corresponding author. The data are not publicly available due to privacy or ethical restrictions.
